# Novel Models of Visual Topographic Map Alignment in the Superior Colliculus

**DOI:** 10.1371/journal.pcbi.1005315

**Published:** 2016-12-27

**Authors:** Ruben A. Tikidji-Hamburyan, Tarek A. El-Ghazawi, Jason W. Triplett

**Affiliations:** 1 School of Engineering and Applied Science, George Washington University, Washington, DC, United States of America; 2 Center for Neuroscience Research, Children’s National Health System, Washington, DC, United States of America; Queen’s University, CANADA

## Abstract

The establishment of precise neuronal connectivity during development is critical for sensing the external environment and informing appropriate behavioral responses. In the visual system, many connections are organized topographically, which preserves the spatial order of the visual scene. The superior colliculus (SC) is a midbrain nucleus that integrates visual inputs from the retina and primary visual cortex (V1) to regulate goal-directed eye movements. In the SC, topographically organized inputs from the retina and V1 must be aligned to facilitate integration. Previously, we showed that retinal input instructs the alignment of V1 inputs in the SC in a manner dependent on spontaneous neuronal activity; however, the mechanism of activity-dependent instruction remains unclear. To begin to address this gap, we developed two novel computational models of visual map alignment in the SC that incorporate distinct activity-dependent components. First, a Correlational Model assumes that V1 inputs achieve alignment with established retinal inputs through simple correlative firing mechanisms. A second Integrational Model assumes that V1 inputs contribute to the firing of SC neurons during alignment. Both models accurately replicate *in vivo* findings in wild type, transgenic and combination mutant mouse models, suggesting either activity-dependent mechanism is plausible. *In silico* experiments reveal distinct behaviors in response to weakening retinal drive, providing insight into the nature of the system governing map alignment depending on the activity-dependent strategy utilized. Overall, we describe novel computational frameworks of visual map alignment that accurately model many aspects of the *in vivo* process and propose experiments to test them.

## Author Summary

In order to process sensory stimuli, precise connections must be established between sensory neurons during development. In the visual system, many connections are organized topographically, such that neighboring neurons monitor adjacent regions of space. In the superior colliculus (SC), converging topographic inputs must be aligned with one another to facilitate integration and preserve the spatial order of the visual scene. In this paper, we propose two novel computational models to describe the alignment of visual inputs in the SC. We demonstrate that both models are able to replicate experimental data obtained from wild type and mutant animals. Interestingly, each model performed differently in response to hypothetical experiments, suggesting they could be differentiated empirically. Thus, we put forth testable models of visual map alignment in the SC and propose experiments to determine which may be used during development.

## Introduction

Processing sensory information is a critical task of the central nervous system, requiring the establishment of precisely ordered synaptic connectivity during development. In the visual system, image-forming regions are organized into topographics maps, such that neighboring neurons monitor adjacent regions of visual space [1, 2]. The development of topographic connections in the visual system has been the focus of intense study, both experimentally and theoretically, elucidating general principles underlying neural circuit wiring [3, 4]. However, these studies have focused primarily on the mechanisms by which topographic connectivity is established for a single projection. In regions that integrate visual information, multiple converging inputs must establish topography and be aligned with one another to facilitate integration [5]. Yet, little is known about the mechanisms by which topographic maps of space are aligned in these regions, in part due to a lack of computational frameworks that model this process.

The superior colliculus (SC) is a critical multisensory integration center that receives visual, somatosensory, and auditory inputs that inform goal-directed head and eye movements [6–8]. The SC receives visual inputs from retinal ganglion cells (RGCs) and Layer 5 pyramidal neurons in the primary visual cortex (V1) [9]. Each of these inputs projects to distinct, but overlapping, sublaminae of the superficial SC, where they are organized topographically and in alignment with one another [10]. The mapping of retinocollicular projections occurs during the first postnatal week in mice, and a combination of molecular cues [11–17], correlated neuronal activity [18–20] and competition [21, 22] have been demonstrated to regulate the establishment of precise retinocollicular topography.

The mechanisms by which V1 inputs establish topography and alignment with retinal inputs are less clear. Mapping of V1 corticocollicular inputs occurs during the second postnatal week in mice, after retinocollicular topography has been established. Previously, we demonstrated that retinal input instructs the alignment of V1 axons in a manner dependent on the normal pattern of spontaneous activity [23]. Subsequent studies confirmed that correlated spontaneous activity originating in the retina propagates throughout V1 and the SC *in vivo* [24], supporting its possible role as the instructive cue for alignment. Further, the timing of spiking acitivty in V1 and the SC is consistent with activity-dependent visual map alignment [25]. However, the underlying mechanisms of activity-dependent alignment remain unclear.

Theoretical modeling of neural circuit development is a powerful tool to both better describe complicated processes and generate novel hypotheses regarding circuit wiring [26]. Indeed, several mathematical models have been developed to describe topographic mapping of retinocollicular projections [27–32]. However, each of the current models has weaknesses and cannot replicate the full complement of empirical data obtained from *in vivo* studies of mutant mice [4]. Further, no theoretical models of visual map alignment have been developed, hindering our ability to probe potential mechanisms of this critical developmental event.

Here, we describe two novel models of visual map alignment in the SC, each of which utilizes a different activity-dependent mechanism for visual map alignment, providing an *in silico* platform to investigate strategies used *in vivo*. First, a Correlational Model assumes that SC neuron firing is driven only by RGC inputs. In this case, alignment of V1 inputs is guided by simple correlation between V1 axon activity and RGC-driven SC activity. Second, an Integrational Model assumes that V1 inputs can drive firing of SC neurons in addition to RGCs. Under these conditions, alignment is driven by weighted integrated activity of both RGCs and V1 inputs. Importantly, both models replicated with high fidelity visual map alignment as observed in wild type (WT) conditions, as well as that observed in transgenic and knockout mouse models. Interestingly, the models could be differentiated *in silico*, as they predicted different behaviors when the retinal drive component was weakened under transgenic model conditions. Based on these findings, we conclude that either correlational or integrational mechanisms may be utilized to achieve visual map alignment, suggest *in vivo* experiments that may be able to distinguish between the two, and speculate on the potential biological advantages of each.

## Results

### Models of visual map alignment in the SC

**Fig 1:**
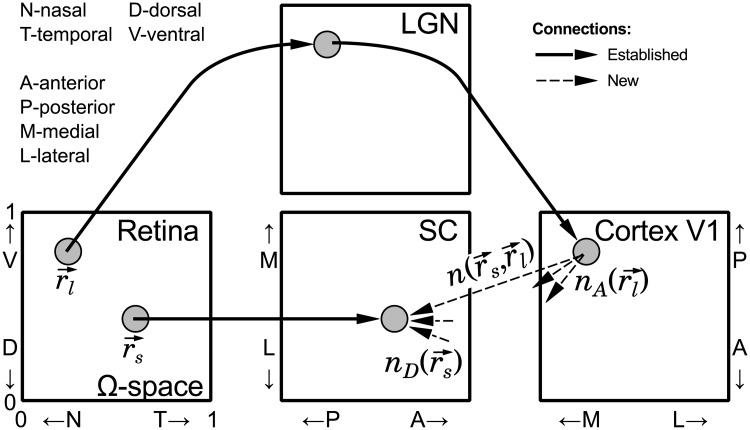
General schematic of topographic maps and coordinate system. Topographic order is established by retinal inputs to the superior colliculus (SC) and the lateral geniculate nucleus (LGN), as well as by LGN projections to visual cortex (V1), at the beggining of the second postnatal week (thick solid arrows). The nasal-temporal (N-T) axis of the retina is mapped along the posterior-anterior (P-A) axis of the SC and medial-lateral (M-L) axis of V1. And, the dorsal-ventral (D-V) axis of the retina is mapped along the lateral-medial (L-M) axis of the SC and anterior-posterior (A-P) axis of V1. New corticocollicular projections (dashed thin arrow) are established during the second postnatal week. These inputs are modeled as a number of connections ($n({\vec{r}}_{s},\;{\vec{r}}_{l})$) between axons originating from neurons located at ${\vec{r}}_{l}$ coordinate in V1 and dendrites of neurons at ${\vec{r}}_{s}$ coordinate in SC. Any location in the SC and V1 are described as vectors (${\vec{r}}_{s}$ and ${\vec{r}}_{l}$, correspondingly) in the same two-dimensional retinal Ω-space. Ω-space is constructed upon mapping in wild type mice and is normalized to unit size along both the N-T and D-V axes of the retina.

In the present study we develop two novel models of visual map alignment in the SC, specifically focusing on the projection from V1 to the SC, which develops during the second postnatal week in mice [23]. We assume that retinocollicular and retino-geniculo-cortical connections have been established during the first postnatal week [33–35], i.e. topography has been established by RGCs in the SC, and V1 neurons are projecting axons from a topographically ordered region (Fig 1). Without a loss of generality, we utilize a common coordinate system based on retinal space to describe the topographic organization in the SC and V1, which allows us to avoid ambiguity when map alignment is not one-to-one, for example in cases of mutant mice. That is, any location in the SC or V1 is a vector $\vec{r}$ in two-dimensional Ω-space, normalized to unit size, associated with the corresponding retinal location (see Fig 1). More specifically, two two-dimensional maps $\Phi :\;{\vec{r}}_{R}\;\to \;{\vec{r}}_{SC}$ and $\Psi :\;{\vec{r}}_{R}\;\to \;{\vec{r}}_{V1}$, are representations of retinal inputs from location ${\vec{r}}_{R}$ into the SC (${\vec{r}}_{SC}$) and into V1 (${\vec{r}}_{V1}$), correspondingly. In order to make direct comparisons to *in vivo* anatomical data, retinal space is projected onto the appropriate axes in both the SC and V1. Specifically, the nasal-temporal (N-T) axis of the SC projects along the posterior-anterior (P-A) axis of the SC and is represented along the medial-lateral (M-L) axis of V1, whereas the dorsal-ventral (D-V) axis of the retina projects along the L-M axis of the SC and is represented along the A-P axis of V1. It is important to note that Ω-space is designed for WT mice, and all projection distortions caused by genetic manipulation are part of the models. In simulations, we study Ω-space in a regular grid where the retina, SC and V1 are represented as two-dimensional layers of 100x100 neurons.

The models present new corticocollicular inputs in SC as a number of connections/synapses (*n*) between axons originating from a given cortical location ${\vec{r}}_{l}\;\in \Omega ,\;\Psi^{-1}:\;{\vec{r}}_{V1}\;\to \;{\vec{r}}_{l}$ and dendrites of SC neurons located in ${\vec{r}}_{s}\;\in \;\Omega ,\;\Phi^{-1}:\;{\vec{r}}_{SC}\;\to \;{\vec{r}}_{s}$. This number is a vector function $n({\vec{r}}_{s},\;{\vec{r}}_{l})$, which is simulated as a four-dimensional array.

To model the development of corticocollicular connections, we extended a stochastical model [22] that was developed to model the establishment of retinocollicular topography and showed best qualitative assessment against experimental data [4]. As in the original approach, the model minimizes total energy *E* in the V1-SC system, which is a function of connectivity. For both models we consider total energy as a sum of chemoaffinity energy (*E*_*a*_), axonal competition energy (*E*_*c*_) and activity-dependent energy (*E*_*u*_):

$$E=E_{a}+E_{c}+E_{u}$$ (1)

The minimum of total energy *E* represents the most stable configuration of corticocollicular connections. We used a modified simulated annealing algorithm, described in [22], to find the minimum of total energy (see Methods section).

Both models share the same representations for the chemoaffinity and competition energies, as described in [22, 36] with minor modifications, but differ in the representation of activity-dependent energy. However, it is important to note that in both cases, the activity-dependent energy function in our model is different from those used for modeling retinocollicular development. Two descriptions for activity-dependent energy reflect different assumptions in model definitions. The first model is based on the assumption that new synapses of V1 axons onto SC neurons are significantly weaker than established synapses with RGCs; therefore this model considers only correlation between activity of SC neurons driven by retinal inputs and V1 neurons. We refer to this model as the “**Correlational**” model. In the second model, we assume that SC neurons integrate activity of both RGC and V1 inputs and that the effect of V1 inputs is not negligible, which we refer to as the “**Integrational**” model. All components for each model are described below.

#### Chemoaffinity energy

We adopted the description of chemoaffinity energy from a model of the development of retinocollicular projections [36]. The relative expression patterns of *EphA*|*EphB* and *ephrin* − *A*|*ephrin* − *B* molecules in orthogonal gradients is similar in V1 as in the retina [9]; therefore the same motivation for formulation of chemoaffinity energy as in [36] can be applied.

$$E_{a}=\sum_{{\vec{r}}_{s}\in \Omega }\sum_{{\vec{r}}_{l}\in \Omega }n\left({\vec{r}}_{s},{\vec{r}}_{l}\right)\left(\alpha_{a}e^{r_{xs}-1}e^{-r_{xl}}-\beta_{a}e^{r_{ys}-1}e^{r_{yl}-1}\right)$$ (2)

where *r*_*xs*_ and *r*_*ys*_ are N-T and D-V components of vector ${\vec{r}}_{s}$; similarly *r*_*xl*_ and *r*_*yl*_ are components of ${\vec{r}}_{l}$; *α*_*a*_ and *β*_*a*_ are model parameters. The examples for the distribution of chemoaffinity energy in SC for neurons located in 9 different positions in V1 are shown in S1 Fig.

#### Competition energy

We assume that cortical axons have the same mechanism to compete for space in the target as the axons from RGCs; therefore, the same motivation for competition energy as in [22] can be applied. Here we use the same formal description as in [22]:

$$E_{c}=\sum_{{\vec{r}}_{l}\in \Omega }\left[\beta_{c}n_{A}^{2}\left({\vec{r}}_{l}\right)-\alpha_{c}\sqrt{n_{A}\left({\vec{r}}_{l}\right)}\right]+\sum_{{\vec{r}}_{s}\in \Omega }\gamma_{c}n_{D}^{2}\left({\vec{r}}_{s}\right)$$ (3)

where *α*_*c*_, *β*_*c*_ and *γ*_*c*_, are model parameters; $n_{A}\left({\vec{r}}_{l}\right)$ is number of axonal connection which originate from V1 neurons located at ${\vec{r}}_{l}$, and $n_{D}\left({\vec{r}}_{s}\right)$ is number of dendritic connections which a SC neuron receives at location ${\vec{r}}_{s}$ (see Fig 1).

#### Activity-dependent energy for correlational model

In the Correlational model, we assume that activity-dependent energy follows from standard Hebbian rules [37]; therefore, energy *E*_*u*_ decreases with increasing numbers of connections between neurons with correlated activity. This model assumes that SC neurons are solely driven by strong retinal inputs, ignoring an role of V1 input on SC neuron firing. For simulations in WT animals, we assume that retinal waves propagate through both established pathways and therefore correlation in activity between V1 axons and SC neurons can be estimated as an exponential function from distance in Ω-space:

$$E_{u}=-\gamma_{u}\sum_{{\vec{r}}_{s}\in \Omega }\sum_{{\vec{r}}_{l}\in \Omega }n\left({\vec{r}}_{s},{\vec{r}}_{l}\right)e^{-\frac{|{\vec{r}}_{s}-{\vec{r}}_{l}|}{\beta_{u}}}$$ (4)

where $|{\vec{r}}_{s}-{\vec{r}}_{l}|=\sqrt{{\left(r_{xs}-r_{xl}\right)}^{2}+{\left(r_{ys}-r_{yl}\right)}^{2}}$ is standard Euclidean distance, *γ*_*u*_ and *β*_*u*_ are model parameters. Parameter *β*_*u*_ describes a decrease in correlation of neural activity between neurons with an increase in the distance between neurons. This parameter was fitted to experimental data [18], as previously described [36].

In cases of simulations in mutant mice, adjustments were made to the Activity-Dependent Energy function to mimic *in vivo* alterations in activity. Specifically, we did simulations in the previously described *Islet2-EphA3* knock-in mice (*Isl*2^*EphA*3/*EphA*3^) [21] and combination mutiants with mice lacking the *β*2 subunit of the nicotinic acetylcholine receptor *Isl*2^*EpA*3/*EphA*3^ / *β*2^−/−^ In *Isl*2^*EphA*3/*EphA*3^ mice, exogenous expression of EphA3 receptor tyrosine kinase in *Isl*2^+^ RGCs results in a duplication of the retina’s projection along the A-P axis of the SC [21]. Alterations in the Activity-Dependent Energy function were needed to account for the reported inequality of signal amplitude between the two functional maps in the SC of *Isl*2^*EphA*3/*EphA*3^ mice, as assessed by intrinsic signal optical imaging [23]. In *β*2^−/−^ mice, the normal pattern of spontaneous waves of retinal activity are disrupted [18, 38, 39]. As a result, development of topography is altered in V1 and the SC, with asymmetric effects along the azimuth axis [20, 40]. Thus, alterations in the Activity-Dependent Energy function were needed to account for these differences:

$$E_{u}=-\gamma_{u}\sum_{{\vec{r}}_{s}\in \Omega }\sum_{{\vec{r}}_{l}\in \Omega }n\left({\vec{r}}_{s},{\vec{r}}_{l}\right)\left[\alpha_{u}e^{-\sqrt{{\left(\frac{r_{xs}-r_{xl}/2}{\kappa_{u}\beta_{u}}\right)}^{2}+{\left(\frac{r_{ys}-r_{yl}}{\beta_{u}}\right)}^{2}}}+e^{-\sqrt{{\left(\frac{r_{xs}-r_{xl}/2-1/2}{\kappa_{u}\beta_{u}}\right)}^{2}+{\left(\frac{r_{ys}-r_{yl}}{\beta_{u}}\right)}^{2}}}\right]$$ (5)

where parameter *α*_*u*_ is used to account for inequality in A-P axis, and *k*_*u*_ allows us to model asymmetric effects in *β*2^−/−^ type mice. Examples of the distributions of activity-dependent energy in the SC for neurons located in 9 different positions in V1 of *Isl*2^*EphA*3/*EphA*3^ and *Isl*2^*EphA*3/*EphA*3^/*β*2^−/−^ mice are shown in S2 Fig.

#### Activity-dependent energy for integrational model

The Integrational model assumes that both retinal and cortical inputs can drive SC neuron firing. Although strong input from the retina dominates in this model, V1 axons also can drive SC neuron firing. Therefore the Activity-Dependent Energy *E*_*u*_ includes two terms as follows:

$$E_{u}=-\gamma_{u}\sum_{{\vec{r}}_{s}\in \Omega }\sum_{{\vec{r}}_{l}\in \Omega }n\left({\vec{r}}_{s},{\vec{r}}_{l}\right)\left[\frac{\sum_{{\vec{q}}_{l}\in \Omega ,{\vec{q}}_{l}\neq {\vec{r}}_{l}}D\left({\vec{r}}_{s},{\vec{q}}_{l}\right)n\left({\vec{r}}_{s},{\vec{q}}_{l}\right)e^{-\frac{|{\vec{r}}_{l}-{\vec{q}}_{l}|}{\beta_{u}}}}{\sum_{{\vec{q}}_{l}\in \Omega ,{\vec{q}}_{l}\neq {\vec{r}}_{l}}D\left({\vec{r}}_{s},{\vec{q}}_{l}\right)n\left({\vec{r}}_{s},{\vec{q}}_{l}\right)}\;+\;\xi_{u}e^{-\frac{|{\vec{r}}_{s}-{\vec{r}}_{l}|}{\beta_{u}}}\right]$$ (6)

where $D\left(\vec{r},\vec{q}\right)=e^{-{\left(\frac{\vec{r}-\vec{q}}{\sqrt{2}\nu }\right)}^{2}}$ defines the synaptic weight of a contact between an axon from a neuron located at ${\vec{q}}_{l}$ in V1 and a dendrite of an SC neuron located at ${\vec{r}}_{s}$ (see [36] for details). The first term in square brackets (Eq 6) defines the total correlation between each V1 axon originated from neurons in ${\vec{r}}_{l}$ locations and all other axons originated from other locations in V1 (${\vec{q}}_{l}$) normalized by weighted number of connections. The second term in Eq (6) describes the correlation between axons from V1 and axons from the retina in a manner similar to that of the Correlational Model (Eq 4). Parameter *ξ*_*u*_ defines the strength of inputs from the retina as compared to maximal synaptic weight for inputs from V1.

In cases of *Isl*2^*EphA*3/*EphA*3^ and *Isl*2^*EphA*3/*EphA*3^/*β*2^−/−^ mice Activity-Dependent Energy for the Integrational model were modified as follows:

$$\begin{array}{l} E_{u}=-\gamma_{u}\sum_{{\vec{r}}_{s}\in \Omega }\sum_{{\vec{r}}_{l}\in \Omega }n({\vec{r}}_{s},{\vec{r}}_{l})[\frac{\sum_{{\vec{q}}_{l}\in \Omega ,{\vec{q}}_{l}\neq {\vec{r}}_{l}}D({\vec{r}}_{s},{\vec{q}}_{l})n({\vec{r}}_{s},{\vec{q}}_{l})e^{-\frac{\left|{\vec{r}}_{l}-{\vec{q}}_{l}\right|}{\beta_{u}}}}{\sum_{{\vec{q}}_{l}\in \Omega ,{\vec{q}}_{l}\neq {\vec{r}}_{l}}D({\vec{r}}_{s},{\vec{q}}_{l})n({\vec{r}}_{s},{\vec{q}}_{l})}\\ +\xi_{u}\left(\alpha_{u}e^{-\sqrt{{\left(\frac{r_{xs}-r_{xl}/2}{\kappa_{u}\beta_{u}}\right)}^{2}+{\left(\frac{r_{ys}-r_{yl}}{\beta_{u}}\right)}^{2}}}+e^{-\sqrt{{\left(\frac{r_{xs}-r_{xl}/2-1/2}{\kappa_{u}\beta_{u}}\right)}^{2}+{\left(\frac{r_{ys}-r_{yl}}{\beta_{u}}\right)}^{2}}}\right)] \end{array}$$ (7)

Note that we simplify *E*_*u*_ by excluding asymmetry terms from the correlation between V1 axons (first term in square brackets), because it slightly affected model performance.

#### Parameter estimation

We estimated parameters for both models to mimic data published previously (see Table 1). Parameters for chemoaffinity (*α*_*a*_ and *β*_*a*_) and competition (*α*_*c*_, *β*_*c*_ and *γ*_*c*_) energies were chosen to keep 18–25 connections per SC neuron. These parameters are slightly adjusted from those previously published for the stochastical model of retinal map development [22, 36].

**Table 1:** Parameters of Correlational and Integrational Models for *WT*, *Isl*2^*EphA*3/*EphA*3^ and *Isl*2^*EphA*3/*EphA*3^/*β*2^−/−^ mice.

Parameter	Correlational Model	Integrational Model
**Type independent Parameters**		
*α*_*a*_	60	60
*β*_*a*_	90	90
*α*_*c*_	450	450
*β*_*c*_	1	1
*γ*_*c*_	1	1
*ν*_*u*_	–	0.15
*ξ*_*u*_	–	3
*WT*		
*β*_*u*_	0.11	0.11
*γ*_*u*_	20	20
*Isl*2^*EphA*3/*EphA*3^		
*α*_*u*_	0.625	0.625
*β*_*u*_	0.11	0.11
*γ*_*u*_	20	20
*κ*_*u*_	1	1
*Isl*2^*EphA*3/*EphA*3^/*β*2^−/−^		
*α*_*u*_	0.625	0.625
*β*_*u*_	0.231	0.231
*γ*_*u*_	8.7	8.7
*κ*_*u*_	4	4

Parameter *βu* for both models was estimated from a best-fit curve to the correlation index in retinal waves for WT mice [18, 36, 38]. Parameters *ν*_*u*_, *ξ*_*u*_ and *γ*_*u*_ were chosen for robust map convergence in both *WT* and *Isl*2^*EphA*3/*EphA*3^ mice. Parameter *α*_*u*_ and *κ*_*u*_ were obtained from experimental data [23] (supplementary Fig 1B) and [40] (Fig 3I), correspondingly. Parameters *βu* and *γ*_*u*_ were scaled for *β*2^−/−^ mice to fit experimental data [20, 38, 40] (see also S3 Fig).

Both models are robust in the range of 20% parameter variation.

### Both models are able to replicate visual map alignment under wild type, *Isl*2^*EphA*3/*EphA*3^ and *Isl*2^*EphA*3/*EphA*3^/*β*2^−/−^ conditions

#### Visual map alignment under wild type conditions

**Fig 2:**
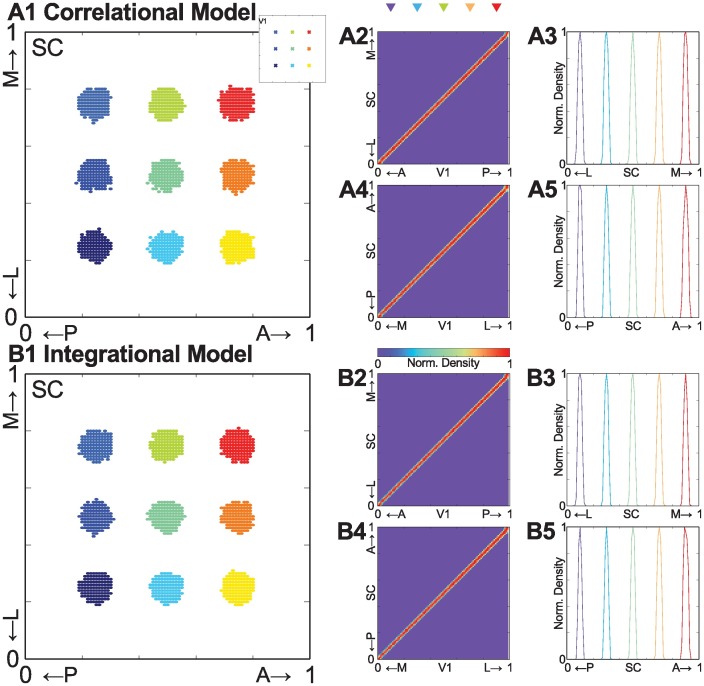
Correlational and Integrational models are able to replicate topographic map alignment under wild type (WT) conditions. **(A1)** and **(B1)** Representative examples of termination zones in the SC from 9 different seed locations of r = 0.04 size in V1 (indicated in A1 inset) for Correlational (A1) and Integrational models (B1). **(A2) & (A4)** and **(B2) & (A4)** One dimensional map of normalized distribution of connection densities along the cardinal axes of V1 and the SC, anterior-posterior (A-P) V1 to medial-lateral (M-L) SC (A2 and B2), L-M V1 to A-P SC (A4 and B4). **(A3) & (A5)** and **(B3) & (A5)** Samples of connection distribution for five different location along A-P (A3 and B3) or L-M (A5 and B5) V1 axis, indicated by colored triangles above the connection density map (A2).

First, we asked if our models could replicate V1 map alignment with the retinal map in the SC under WT conditions. We performed stochastic minimization of global energy function (Eq 1) using a modified simulated annealing algorithm [22, 32, 41] as described in the Methods section. Critically, both models are able replicate visual map alignment under WT conditions (Fig 2). To begin, the connectivity pattern is examined by plotting termination zones in the SC for projections from 9 local neighborhoods in V1 with radius 0.04 (schematically indicated on Fig 2A1 inset). Both models show topographically appropriate patterns of termination zones (Fig 2A1 and 2B1). We then estimated the density of connections and examined normalized density distributions along both axes of the SC (Fig 2A2, 2A4, 2B2 and 2B4). Finally, we sampled 5 connection density distributions and estimated the width of termination zones at 20% of maximum density distribution. The width of a given termination zone is approximately 5% of SC area, which is in agreement with experimental observations [23]. Importantly, both models show a similar speed of convergence to a stable connectivity pattern (S4 Fig), suggesting that the rate of refinement is not dramatically different between activity-dependent mechanisms.

#### Visual map alignment under *Isl*2^*EphA*3/*EphA*3^ conditions

**Fig 3:**
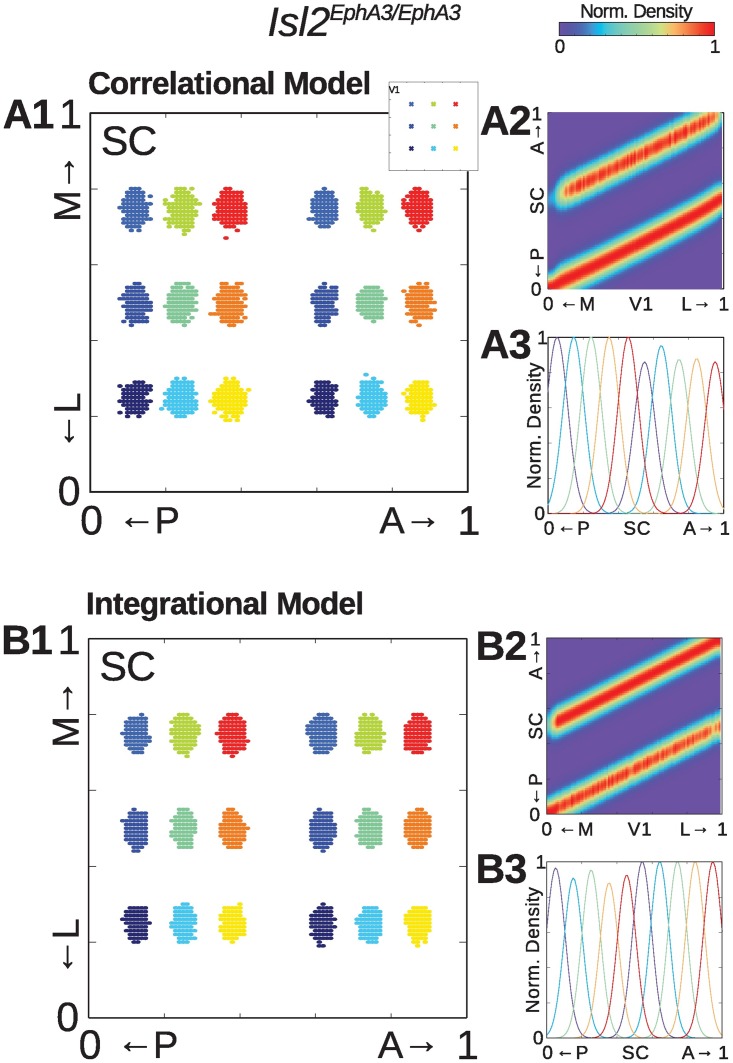
Modeling of visual map alignment under *Isl*2^*EphA*3/*EphA*3^ mutant conditions. **(A1)** and **(B1)** Representative examples of termination zones in the SC from 9 different seed locations in V1 (indicated in A1 inset) for Correlational (A1) and Integrational models (B1) under *Isl*2^*EphA*3/*EphA*3^ conditions. **(A2)** and **(B2)** One dimensional map of normalized distribution of connection densities along the azimuth axes of the medial-lateral (M-L) V1 and the anterior-posterior (A-P) SC. **(A3)** and **(B3)** Samples of connection distribution for five different location along L-M V1 axis.

Next, we asked if our models could replicate map alignment under conditions in which the retinocollicular projeciton is altered, as in *Isl*2^*EphA*3/*EphA*3^ mice. In these mice, the retina’s projection to the SC is duplicated along the A-P axis of the SC, but singular along the L-M axis [21]. And, the V1 projection bifurcates along the A-P axis to maintain alignment with the duplicated retinal map [23]. Strikingly, both models were able qualitatively replicate this experimental observation: axons from the same local neighborhoods in V1 as in Fig 2 project to two locations along the A-P axis of the SC (Fig 3A1 & 3B1). Indeed, examining only projections along the azimuth axis reveals a clear duplication of projections along this axis (Fig 3A2 & 3B2). Importantly, the pattern of distribution along the L-M axis appeared unchanged, consistent with predictions based on intrinsic signal optical imaging [23].

For both Correlational and Integrational models, the width of distrubution along the A-P axis appears similar for V1 axons projecting to the anterior and posterior maps (Fig 3A3 & 3B3). This prediction is in contrast to previous findings demonstrating that terminations zones of V1 axons are signifcantly larger in the posterior domain of *Isl*2^*EphA*3/*EphA*3^ mice [23]. One possible explanation for this mismatch may be alterations in the distance of spatial correlation of activity during development. In *Isl*2^*EphA*3/*EphA*3^ mice, a full representation of azimuth is compressed into approximately half the anatomical territory of the SC. Thus, the distance of relevant correlations may be reduced by half, which in the models would be reflected by changing *κ*_*u*_ to 0.5 for simulations under *Isl*2^*EphA*3/*EphA*3^ conditions. However we did not find any justification for *κ*_*u*_ adjustment in the literature and used parameters as they are stated in Table 1. Regardless, even with our canonical parameters both models are in good qualitative agreement with profiles of anatomical tracing experiments in *Isl*2^*EphA*3/*EphA*3^ mice [23].

#### Visual map alignment in *Isl*2^*EphA*3/*EphA*3^/*β*2^−/−^ mice

Previously, we showed that the normal pattern of spontaneous activity is required for the alignment of visual maps in the SC [23]. Specifically, mice in which the pattern of spontaneous waves are disrupted (*β*2^−/−^) were crossed into the *Isl*2^*EphA*3/*EphA*3^ line. In these combination mutants, the retina’s projection to the SC is still duplicated along the A-P axis of the SC, though each termination zone is broader. However, in contrast to *Isl*2^*EphA*3/*EphA*3^ / *β*2^+/−^ control mice, in which the V1 projection to the SC is duplicated to align with the retinal map, tracings of V1 projections in *Isl*2^*EphA*3/*EphA*3^/*β*2^−/−^ mice result in only a single broad termination zone.

**Fig 4:**
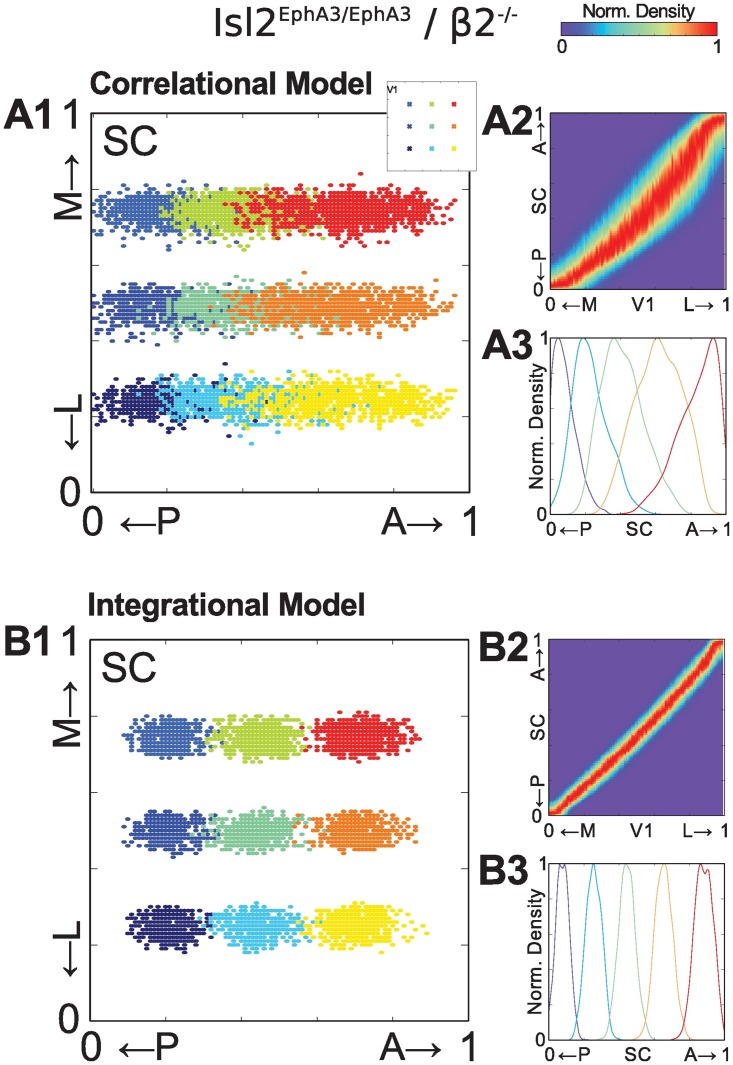
Modeling of visual map alignment under *Isl*2^*EphA*3/*EphA*3^/*β*2^−/−^ mutant conditions. **(A1)** and **(B1)** Representative examples of termination zones in the SC from 9 different seed locations in V1 (indicated in A1 inset) for Correlational (A1) and Integrational models (B1) under *Isl*2^*EphA*3/*EphA*3^/*β*2^−/−^ conditions.**(A2)** and **(B2)** One dimensional map of normalized distribution of connection densities along the azimuth axes of the medial-lateral (M-L) V1 and the anterior-posterior (A-P) SC. **(A3)** and **(B3)** Samples of connection distribution for five different location along L-M V1 axis.

We next asked if our models were able to replicate this key finding under *Isl*2^*EphA*3/*EphA*3^/*β*2^−/−^ conditions. To do this the parameters of the *Isl*2^*EphA*3/*EphA*3^ model were adjusted to reflect experimental observations regarding the distance between neurons with correlated activity patterns in *β*2^−/−^ mice [20, 38, 40] (see Table 1). Strikingly, we found that in both models, V1 projects to a single map along both the A-P and L-M axes of the SC under *Isl*2^*EphA*3/*EphA*3^/*β*2^−/−^ conditions (Fig 4). Further, both models show an increase in termination zone size, though the increase was dramatically larger for simulations of the Correlational model (Fig 4A) compared to the Integrational model (Fig 4B). This difference reflects the ability of V1 neurons to drive SC neuron firing in the Integrational model, as such locally correlated activity of V1 axons guided by chemoaffinity forces leads to better refinement, though still not as good as under WT conditions. Interestingly, we found no qualitative difference in the speed of convergence between both models, nor better convergence with increased numbers of iterations (S4 Fig), suggesting that differences in refinement were not due to different temporal dynamics. Importantly, the connection densities along the A-P axis for both models (Fig 3C2, 3C3, 3D2 and 3D3) are in qualitative agreement with anatomical tracing experiments performed in *Isl*2^*EphA*3/*EphA*3^/*β*2^−/−^ mice (see [23] Fig. 6H).

Taken together with results of our simulations under WT and *Isl*2^*EphA*3/*EphA*3^ conditions, these findings suggest that both Correlational and Integrational models serve as valid frameworks to investigate visual map alignment in the SC. Unfortunately, the ability of both models to replicate *in vivo* findings under all conditions published precludes our ability to conclude which type of activity-dependent mechanisms of alignment might be utilized during visual map alignment. As such, we next attempted to differentiate between the models in some way, which may inform future *in vivo* experiments aimed at determining the nature of activity-dependent visual map alignment.

### Weakening of retinal inputs to SC reveals distinct behaviors of Correlational and Integrational models

The major distinction between our Correlational and Integrational models is the ability of V1 inputs to drive SC neurons during the process of visual map alignment. In both models, retinal inputs have strong drive, which instructs V1 inputs to align with the retinal map. However, we noted that during some simulations of the Integrational model under any condition, transient clusters of V1 terminals could be observed in the SC. This anecdotal observation suggested that the Correlational and Integrational models might behave differently under conditions in which retinal drive were reduced during visual map alignment. To test this, we performed an *in silico* experiment in which we simulated visual map alignment under *Isl*2^*EphA*3/*EphA*3^ conditions, but with weakened ability of retinal input to drive SC neuron firing.

**Fig 5:**
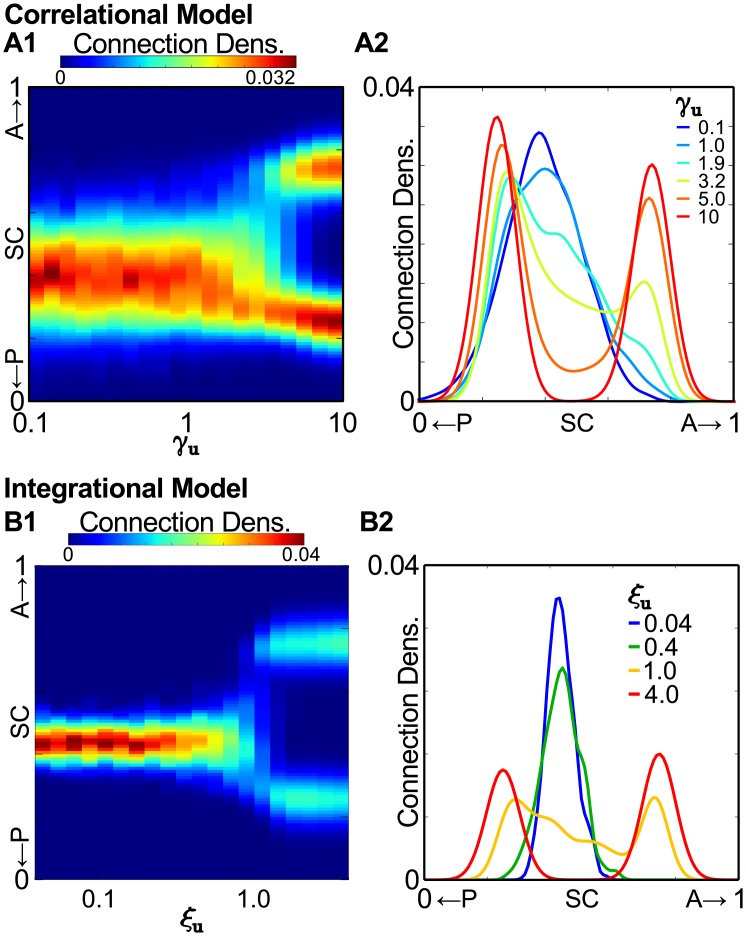
Distinct behaviors of Correlational and Integrational models when retinal drive is weakened. **(A1)** and **(B1)** Connection density plots for V1 axons originating from a central region of the medial-lateral axis of V1 onto the anterior-posterior (A-P) axis of the SC as a function of altering the parameter for retinal drive in Correlational (*γ*_*u*_, A1) and Integrational (*ξ*_*u*_, B1) models. **(A2)** and **(B2)** Samples of connection density along the A-P axis for selected values of *γ*_*u*_ (A2) or *ξ*_*u*_ (B2).

In the Correlational model, weakening retinal drive is equal to a gradual decreasing of *E*_*u*_, which we model by scaling down the *γ*_*u*_ parameter. For this analysis, we simulated the termination patterns of V1 axons projecting from the center of the L-M axis (*r*_*xl*_ = 0.5) under *Isl*2^*EphA*3/*EphA*3^ conditions. As expected, simulations in which retinal drive is similar to previous simulations (e.g. *γ*_*u*_ = 10), projections from V1 are bifurcated into two termination zones along the A-P axis (Fig 5A). And, not surprisingly, when retinal drive is dramatically reduced (e.g. *γ*_*u*_ = 0.1), V1 axons terminate broadly along the A-P axis and only in a single termination zone (Fig 5). Interestingly, the transition was gradual between a single broad termination zone when retinal drive is weak to duplicated termination zones when retinal drive is high. This pattern of change is reminiscent of supercritical pitchfork bifurcation observed in dynamical systems [42], and implies that two termination zones of cortical axons may be a result of bi-stability for individual axons.

In simulations with the Integrational model, we modeled the weakening retinal drive by decreasing the factor *ξ*_*u*_. Similar to observations from the Correlational model, simulations with high retinal drive (e.g. *ξ*_*u*_ = 4) resulted in a bifurcation of V1 projections, while in those with weak retinal drive (e.g. *ξ*_*u*_ = 0.04) a single termination zone was observed. Interestingly, the width of connection densities were not as wide under the latter conditions compared with simulations in the Correlational model, due to the ability of local V1 inputs to drive correlated activity in the Integrational model. Further, we observed that the transition from projections to a single termination zone when retinal drive is weak to duplicated termination zones when retinal drive is strong was much sharper for the Integrational model compared to the Correlational model.

Taken together, these *in silico* experiments suggest that the models can be differentiated. Importantly, the diagrams generated by these experiments are not strictly classical bifurcation diagrams for dynamical systems [42]. Despite this, they reveal characteristic features of the total energy function, which affects the dynamics of connectivity patterns during development. Previously, it was noted that energy functions for competition [22] and for activity-dependence [36] have stable fix points which is an attribute of dynamical systems. Although our simulations should be considered only as optimization procedures, the minimum of energy function and corresponding peaks of connection density, are stable fix points of a dynamical system.

## Discussion

The establishment of precise, topographically-ordered connectivity in the visual system is critical for efficient relay of spatial information. In associative centers of the brain, topographic inputs from multiple areas (and often multiple modalities) must be aligned with one another to facilitate integration. Here, we describe two computational models that simulate the activity-dependent alignment of converging topographic inputs from the retina and V1 in the SC, a critical integrative midbrain nucleus. The first model is based on a strictly correlative mechanism, whereby incoming V1 terminals are stabilized onto neurons in the SC whose activity is driven by RGC inputs that monitor the same region of space. The second model incorporates the ability of V1 inputs to drive SC neuron activity during alignment in addition to RGC drive. Both models qualitatively reflect data derived from empirical experiments in WT, *Isl*2^*EphA*3/*EphA*3^ transgenic, and combination *Isl*2^*EphA*3/*EphA*3^/*β*2^−/−^ mutant mice. These findings suggest that either strategy may be utilized in the developing SC and set the stage for future experiments to distinguish between these mechanisms.

### Distinction of visual map alignment models from retinocollicular mapping models

The development of visual inputs in the SC occurs as a two step process: first, retinocollicular inputs establish topographic order in a manner dependent on molecular cues, correlated neuronal activity and competition during the first postnatal week; second, V1 inputs are instructed by RGCs to terminate in alignment with the retinocollicular map in a manner dependent on spontaneous activity. Thus, both of our computational models of visual map alignment focus on the establishment of topography by V1 neurons and are based on previous stochastic models that describe the development of retioncollicular topography [22, 43]. Based on our previous work demonstrating the importance of correlated spontaneous activity during visual map alignment [23], the most critical component of each model is the activity-dependent energy. For the Correlational model, activity-dependent alignment is achieved via Hebbian “fire together, wire together” rules [37], wherein simple correlations between firing patterns of V1 axons and SC neurons are used. In contrast, the Integrational model considers the possibility that V1 inputs can drive SC neuron firing during visual map alignment.

Although both of the models presented here are based on the stochastic models previously decribed to model retinocollicular development, it is critical to note that the process, and thus the performance, of the models is fundamentally different. When modeling retinocollicular development, the landscape of activity-dependent energy in any given region of the SC is essentially flat prior to simulation, due to the random connectivity. As such, during modeling, newly established connections form the energy profile, progressively developing energy wells in each region as dictated by the local density of RGC inputs, until a stable configuration is achieved. In contrast, when modeling the alignment of V1 inputs in the SC, the landscape of activity-dependent energy is in a pre-defined state by RGC inputs (Eqs 4–7, Supplementary S2 Fig). Indeed, these differences revealed themselves in the behavior of our models during *in silico* experiments performed in which we weakened retinal drive. Under these conditions, the Integrational model performs similar to modeling retinocollicular development, in that activity-dependent energy progressively decreases. Alternativley, in simulations with the Correlational model, which most closely resembles the retinocollicular mapping models on which our alignment models are based, activity-dependent energy can only decrease when retinal drive is sufficient. Understanding the nature of interactions between retinal and V1 inputs during visual map alignment is critical for developing a more robust model of this process.

### Correlational and Integrational models of visual map alignment each replicate experimental data

Importantly, both models replicate *in vivo* findings from WT and mutant animals, though with subtle differing degrees of fidelity. For example, while both models predict that V1 projections will bifurcate to align with a duplicated retinal map under *Isl*2^*EphA*3/*EphA*3^ conditions, neither predicts that the termination zone area of posterior-projecting V1 axons will be larger than anterior-projecting V1 axons, as we previously found [23]. This limitation may derive from innacurate estimation of the distance over which activity is properly correlated in the SC of *Isl*2^*EphA*3/*EphA*3^ mice. On one hand, since an entire azimuth representation is compressed into approximately half the SC, the relevant correlation distance may need to be halved as well. On the other hand, correlations between V1 and SC activities may actually be correlated over larger distances in *Isl*2^*EphA*3/*EphA*3^ mice, since two locations separated by a significant distance will fire with similar timing. Further, it remains unclear why only one of the two termination zones of V1 axons in *Isl*2^*EphA*3/*EphA*3^ does not refine as well as those observed in WT animals. It may be related to the differences in subtypes of RGCs that project to each domain [44], given that distinct subtypes may participate differently during spontaneous retinal waves [45]. Elucidation and incorporation of these parameters of spontaneous activity into future models is necessary to overcome the limitations of our current models.

Another limitation of these models is the underlying assumption that the representation of visual space in each region is symmetrical, which does not accurately reflect anatomical and functional data. Indeed, in several species, portions of the visual field are over-represented in the retina, V1 and the SC. In the mouse visual system, which these computational frameworks are meant to model, RGC density is highest centrally with a slight ventral bias (i.e. upper visual field) and decreases with eccentricity [46]. Similarly in both V1 and the SC, the central visual field is over-represented [20, 47]. However, in other species, the asymmetric representation of visual space can differ between regions. For instance, in the macaque, lower visual field is over-represented in V1 [48], while upper visual field is over-represented in the SC.

How might alignment be achieved in such a situation and could our models account for this? While we did not model this directly, possible distortions of symmetry, such as expansions and contractions, are included in the Φ and Ψ functions and, thus, are implicit to the model. However, the pliability of such distortions are limited by the competition energy component of our models, and, therefore, these models may not be ideal for investigating more drastic “sign reversals.” Application of our models in these contexts may have to incorporate changes in competition energy. Another caveat is that our models deal strictly with development, where we model the pattern of spontaneous activity driving alignment to influence all regions of the retinotopic map uniformly. However, if non-uniform, experience-dependent changes drive differences in asymmetry between regions, then distinct mechanisms, and thus models, may be needed to describe this process.

It is also critical to note that these models focus solely on the alignment of excitatory inputs from the retina and V1 onto excitatory principal cells of the superficial SC, ignoring putative connections with inhibitory populations. Indeed, the SC is densely packed with inhibitory neurons that modulate both the response to visual stimuli and the sensorimotor transformation to saccadic eye movements [49, 50]. However, while GABAergic synapses are present in the SC during the period of retinocollicular map formation and visual map alignment [51], they are weak and their role in either process is not clear. Regardless, inclusion of the development of connections between V1 neurons and inhibitory inputs in the SC, as well as lateral connections within the SC, would make for a more robust model.

### Distinction betweeen Correlational and Integrational models in *in silico* tests

In order to distinguish the Correlational and Integrational models from one another, we leveraged the duplicated map of azimuth in *Isl*2^*EphA*3/*EphA*3^ to perform a modified bifurcation analysis. To do so, we performed simulations with both models in which we varied the parameter relating to the strength of retinal drive (*γ*_*u*_ for Correlational and *ξ*_*u*_ for Integrational). For the Correlational model, we found that increasing retinal drive led to a gradual transition from a single, broad map to a sharply tuned duplicated map. The shape of this curve was strikingly similar to that of the supercritical pitchfork bifurcation associated with dynamical systems, albeit a static version rooted in a spatial domain.

The behavior of the Integrational model to increasing retinal drive under *Isl*2^*EphA*3/*EphA*3^ conditions was strikingly different. Here, the transition from single to duplicated map was sharp, and suggestive of multistability within the system. Interestingly, we previously found that the retinocollicular map in heterozygous *Isl*2^*EphA*3/+^ mice can be organized in one of three possible ways [43], reminiscent of the either/or prediction of the Integrational model observed here. Together, these findings suggest the possibility that the development of topography in general may observe the rules of multistable systems.

In general the Integrational model is more robust to variation of retinal input strength. It shows smaller variance in alignment accuracy to a broader range of retinal input strengths (Fig 5B), which may be considered as potential biological advantage. In contrast weakening retinal inputs below some threshold gradually distorts topographic map alignment in the Correlational model (Fig 5A). Therefore, the *in silico* tests performed here on simplified computational models of a complex biological process are severely limited in their predicitive powers. Further, our data do not favor conclusively either the Correlational or Integrational model and more data are needed to determine if either is a valid representation of *in vivo* processes.

### Differentiation of Correlational and Integrational models *in vivo*

Given that both models are able to replicate the limited *in vivo* data from mutant animals, the question of which is utilized remains unresolved. An exploration of the biological advantages of each may point towards which mechanism might be utilized. On one hand, the Correlational model might be energetically favorable compared to the Integrational model, since developing V1 inputs do not need to invest in expressing the full complement of pre-synaptic machinery at each transient early contact. Additionally, one might imagine that use of a correlational mechanism might lead to faster refinement, again making it more energetically favorable. However, our *in silico* modeling does not indicate that the Correlational model resolves to a steady state faster than the Integrational model (S4 Fig), though this is not necessarily representative of the speed of refinement *in vivo*. On the other hand, the energy investment required to execute the Integrational model may confer other advantages to the development of visual circuitry in the SC. For instance, multiple subtypes of visual neurons are found in the SC [52], and the ability of V1 inputs to contribute to SC neuron firing during development may help to ensure that they integrate into the appropriate sub-circuit. In support of this possibility, recent evidence suggests that fine-grain topography in the SC may be sacrificed to allow for the establishment of microdomains of neurons tuned to the same aspect of the visual scene [53]. However, critical aspects of the nature of the developing circuitry in the SC remain unknown, preventing us from favoring one model over the other.

One key piece of evidence that might distinguish these models relates to the distinction between the two formulations: namely, whether V1 inputs can drive SC neuron firing during development. Electron microscopy studies indicate that V1 inputs form synaptic contacts onto SC cells during development, which mature over time [54]. However, to our knowledge no study has explored the physiological characteristics of corticocollicular inputs throughout development, perhaps due to the circuitous route from V1 to the SC preventing the isolation of the preserved tract in a slice. A potential alternative may be to leverage the power of optogenetics to expresses light-excitable channels in V1 during development. Slices could then be made of the SC and the termials of corticocollicular afferents stimulated while recoding from SC neurons. Understanding the potency of V1 inputs over the course of visual map alignment would provide substantial insight to the mechanisms underlying this critical event, as well as inform the development of more accurate models of visual map alignment.

### Conclusion

Here we have described two novel computational models of the development of alignment between retinal inputs and those from V1 in the SC. The major difference between the models relates to the mechanism of activity-dependent refinement. The models behave differently in *in silico* experiments in which retinal drive the SC is weakened during simulations, suggesting differences in the nature of map alignment depending on the mechanism of activity-dependent refinement. Overall, the Correlational and Integrational frameworks presented here accurately model known aspects of visual map alignment, but further experimentation is needed to determine which of the activity-dependent mechanisms is utilized *in vivo*.

## Methods

### Optimization process for find minimum of the energy function

A modified simulated annealing algorithm [22] was used to find the minimum of energy function (Eq 1). For each neuron in the 100x100 grid, the algorithm produces 15,000 steps, 150,000,000 steps in total. At each step, the algorithm adds one connection and removes another one randomly. The probability to accept or reject addition or removal of a connection is modeled by the sigmoid function from changing in in total energy (Δ*E*) as followed:

$$P=\frac{1}{1+e^{4\Delta E}}$$ (8)

Initially connections are randomly distributed such that each neuron receives 50 connections on average. We also tested our models under two extreme initial conditions: totally disconnected and all-to-all connected networks. No variation in results were found under either condition.

We confirmed that 150,000,000 steps are enough by performing a simulation when number of steps was doubled. Neither model, under any parameter set, achieved better convergence with double the number of steps (S4 Fig). Therefore, we conclude that 150,000,000 steps allow our algorithms to reach steady-state energy minimums.

### Model realization and source code

The modified simulated annealing algorithm was implemented in Cython computer language with Python wrapper. We used the Python numerical library (numpy) and GNU scientific library (gsl) for random number generation, matrix manipulations and operation vectorization. One optimization procedure for the Correlational model requires on average 10 hours of single processor time, while the Integrational model needs approximately 16 hours of single processor time. Source code on the model and required scripts will be made public available via ModelDB website after publication (https://senselab.med.yale.edu/ModelDB/showModel.cshtml?model=195658).

### Parameters space study

We studied the robustness of parameters to variation, as well as general model behavior, in a wide range of parameter space, which was estimated to require around 1.3 years of simulation time on four cores of a desktop computer. In this study, we exploited embarrassingly parallel computing on 1344 cores of a high performance Cray XE6/XK7 cluster to speed up computations to one week.

### Post-modeling analysis

A connectivity four-dimensional array (*n*) was sampled to verify one dimension mapping. To obtain connectivity density, standard Silverman method [55] implemented in the Python scientific library (scipy) was used.

## Supporting Information

S1 Fig Examples of the distributions of chemoaffinity energies in SC. 2D heat-maps of the chemoaffinity energy Eq (2) for axons of neurons located at 9 different places in V1. Each plot shows the distributions of chemoaffinity energies for neurons in one location in V1. Location from left to right and top to down are: (0.25, 0.25) (0.25, 0.5) (0.25, 0.75), (0.5, 0.25) (0.5, 0.5) (0.5, 0.75), (0.75, 0.25) (0.75, 0.5) (0.75, 0.75).
(JPG)

S2 Fig Examples of the distributions of activity-dependent energies in SC for *Isl*2^*EphA*3/*EphA*3^ and *Isl*2^*EphA*3/*EphA*3^ / *β*2^−/−^ mice. 2D heat-maps of the activity-dependent energy, Eq (5), under *Isl*2^*EphA*3/*EphA*3^ conditions (**Top**) and *Isl*2^*EphA*3/*EphA*3^ / *β*2^−/−^ conditions (**Bottom**). In both sets, each of 9 plots shows distribution of activity-dependent energy for neurons in one location in V1. Locations are the same as in S1 Fig.
(JPG)

S3 FigFitting parameters of spatial correlation in retinal waves for *WT* and *β*2^−/−^ mice.**Black** and **Gray** are experimental data of activity correlation index in retinal waves with distance in *WT* and *β*2^−/−^ mice from [38]. **Red** and **Blue dashed** lines are exponential functions $a+be^{-\frac{x}{k}}$ fitted to experimental data. The ratios *b*_*WT*_/*b*_*β*2*K*0_ and *k*_*WT*_/*k*_*β*2*K*0_ were used to scale *γ*_*u*_ and *β*_*u*_ parameters, correspondingly, in *β*2^−/−^ mice model (see Table 1).(JPG)

S4 Fig Convergence of the models. Example of convergence for each model in Wild Type (*WT*, **A**) and two transgenic mice (*Isl*2^*EphA*3/*EphA*3^,**B** and *Isl*2^*EphA*3/*EphA*3^/*β*2^−/−^,**C**) are shown. Each graph in rows **1** and **2** is the distribution of connection density along the A-P axis of the SC for 5 locations along the L-M axis in V1. Color coding is the same as in Figs 2**A5** and 2**B5**, 3**A3** and 3**B3**, 4**A3** and 4**B3**. Graphs in each row correspond to initial conditions (left-most graph) and after the indicated number of iterations. **A3**,**B3**,**C3**: Plot of the Euclidean distance in multi-dimensional space between distributions shown in A1, A2, B1, B2, C1, and C2 as a function of iteration number (sampling every 500,000 steps). The number of steps is plotted logarithmically on the x-axis, and the number of steps utilized for analysis of map organization (150,000,000 steps) is indicated by a black triangle.
(JPG)
