# Supplementary figures and images for: Novel Models of Visual Topographic Map Alignment in the Superior Colliculus
Source: PLoS Comput Biol. 2016 Dec 27;12(12):e1005315. doi: 10.1371/journal.pcbi.1005315 (PMC5226834; doi:10.1371/journal.pcbi.1005315)

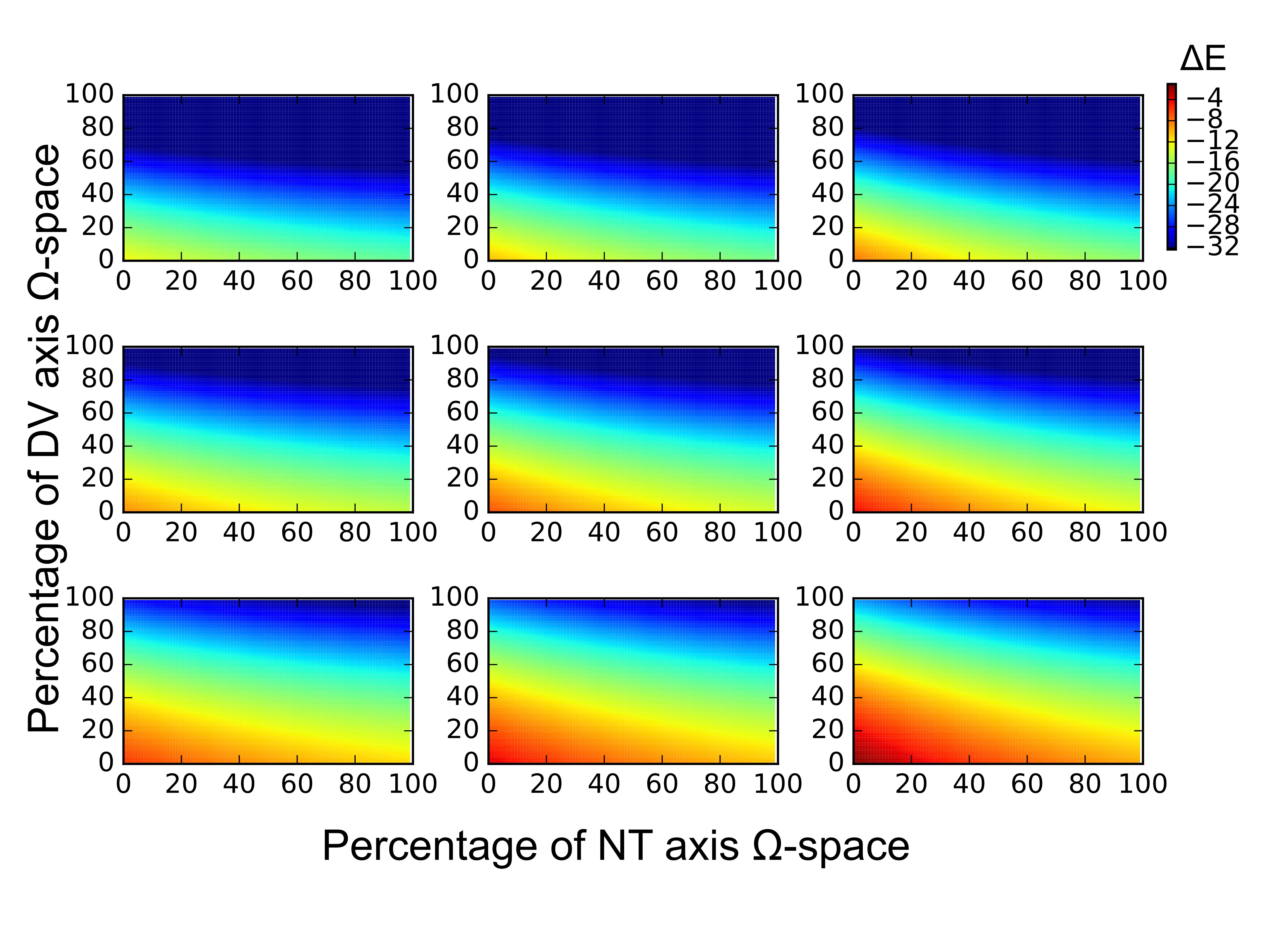

Supplement: S1 Fig — 2D heat-maps of the chemoaffinity energy Eq (2) for axons of neurons located at 9 different places in V1. Each plot shows the distributions of chemoaffinity energies for neurons in one location in V1. Location from left to right and top to down are: (0.25, 0.25) (0.25, 0.5) (0.25, 0.75), (0.5, 0.25) (0.5, 0.5) (0.5, 0.75), (0.75, 0.25) (0.75, 0.5) (0.75, 0.75). (JPG) [file pcbi.1005315.s001.jpg]

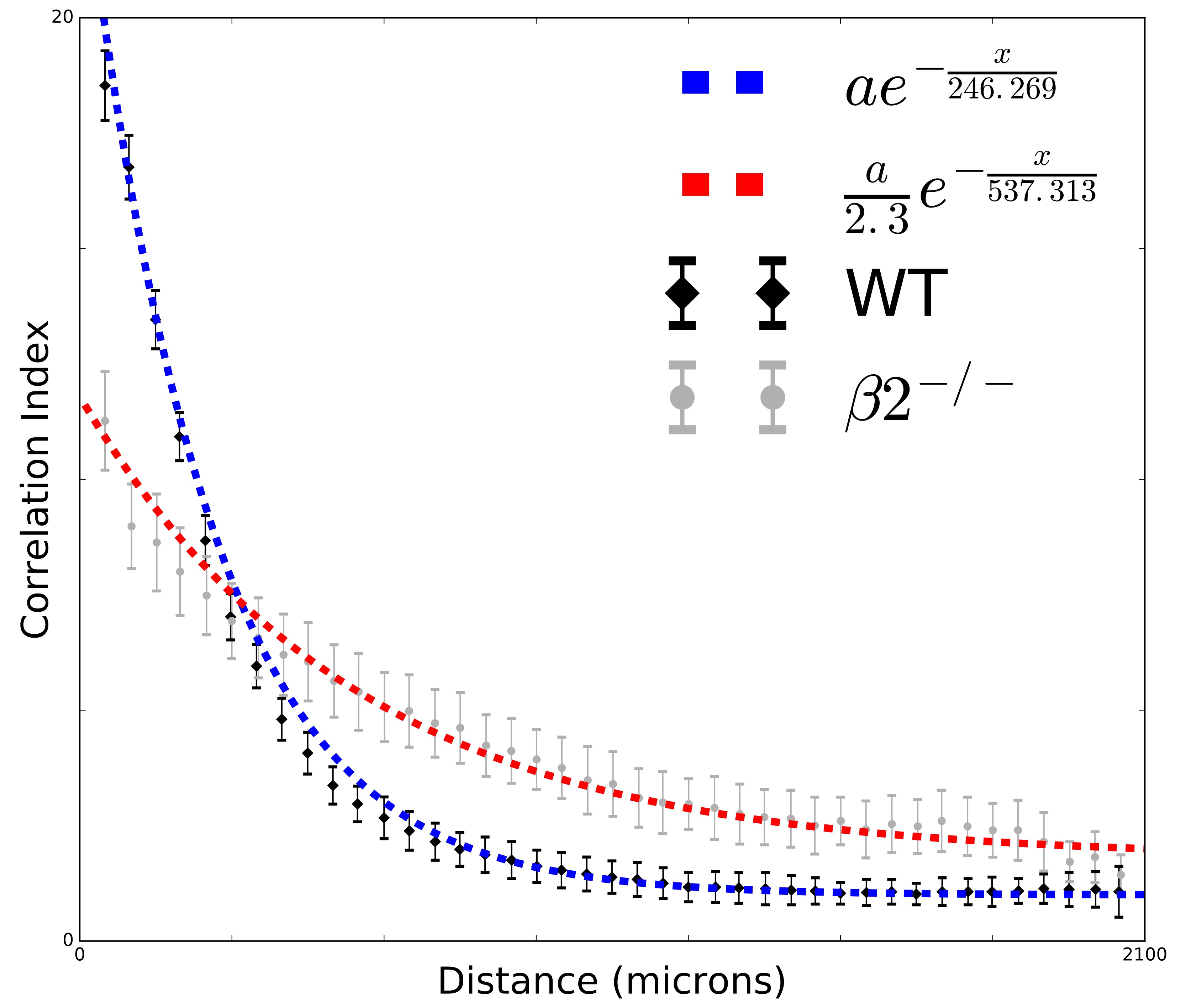

Supplement: S3 Fig — Black and Gray are experimental data of activity correlation index in retinal waves with distance in WT and β2−/− mice from [38]. Red and Blue dashed lines are exponential functions a+be-xk fitted to experimental data. The ratios bWT/bβ2K0 and kWT/kβ2K0 were used to scale γu and βu parameters, correspondingly, in β2−/− mice model (see Table 1). (JPG) [file pcbi.1005315.s003.jpg]

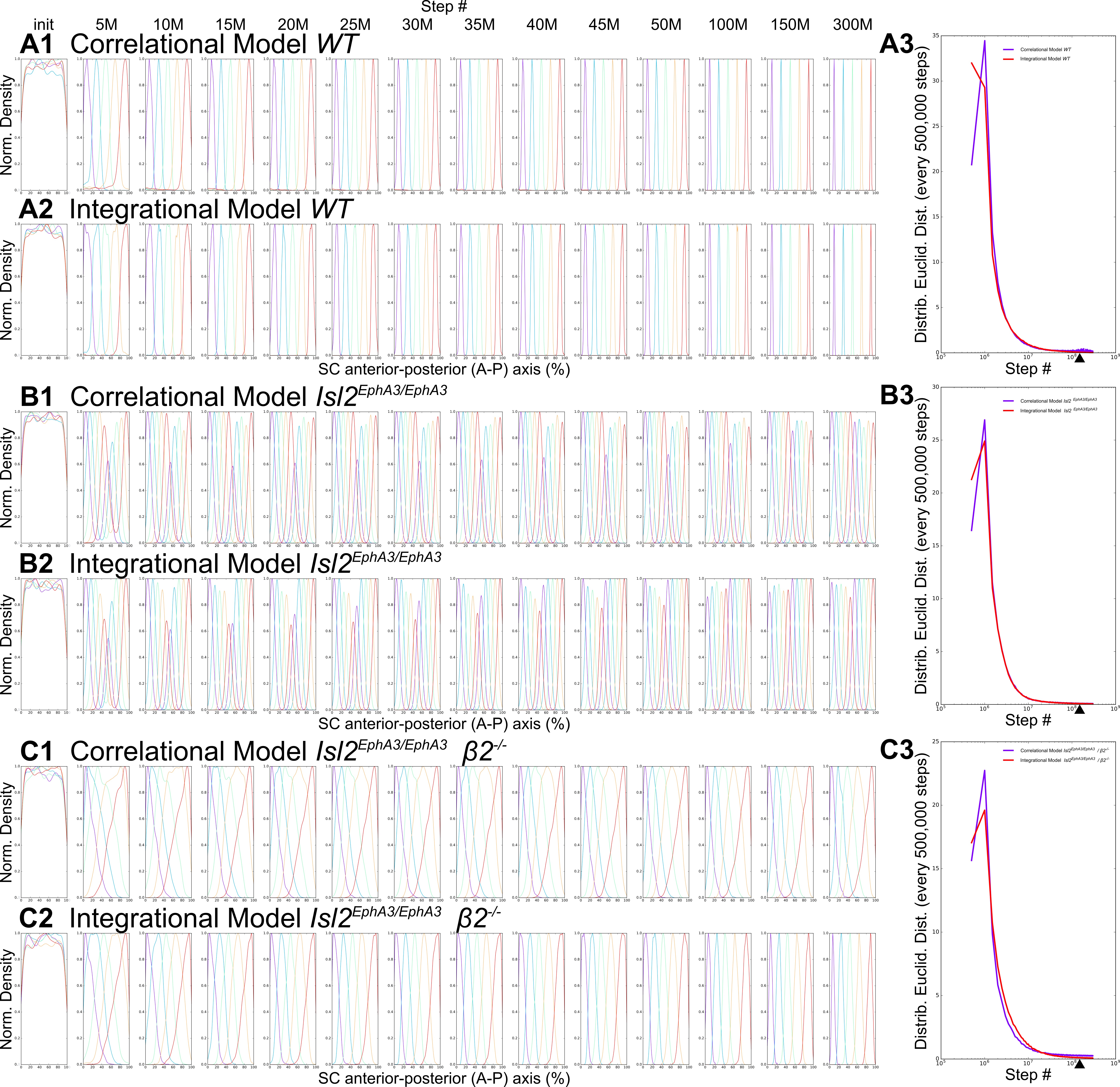

Supplement: S4 Fig — Example of convergence for each model in Wild Type (WT, A) and two transgenic mice (Isl2EphA3/EphA3,B and Isl2EphA3/EphA3/β2−/−,C) are shown. Each graph in rows 1 and 2 is the distribution of connection density along the A-P axis of the SC for 5 locations along the L-M axis in V1. Color coding is the same as in Figs 2A5 and 2B5, 3A3 and 3B3, 4A3 and 4B3. Graphs in each row correspond to initial conditions (left-most graph) and after the indicated number of iterations. A3,B3,C3: Plot of the Euclidean distance in multi-dimensional space between distributions shown in A1, A2, B1, B2, C1, and C2 as a function of iteration number (sampling every 500,000 steps). The number of steps is plotted logarithmically on the x-axis, and the number of steps utilized for analysis of map organization (150,000,000 steps) is indicated by a black triangle. (JPG) [file pcbi.1005315.s004.jpg]
